# Two-Antenna Gain Measurement Method Using Two UAVs

**DOI:** 10.3390/s26072174

**Published:** 2026-03-31

**Authors:** Venkat Reddy Kandregula, Zaharias D. Zaharis, Evangelos Vassos, Qasim Z. Ahmed, Faheem A. Khan, William G. Whittow, Thomas Whittaker, Pavlos I. Lazaridis

**Affiliations:** 1 School of Computing and Engineering, University of Huddersfield, Huddersfield HD1 3DH, UK; q.ahmed@hud.ac.uk (Q.Z.A.); f.khan@hud.ac.uk (F.A.K.); p.lazaridis@hud.ac.uk (P.I.L.); 2 School of Electrical and Computer Engineering, Aristotle University of Thessaloniki, 54124 Thessaloniki, Greece; zaharis@auth.gr; 3 RF & Microwave Engineering, Steatite Antennas, Leominster HR6 0QF, UK; evangelos.vassos@steatite.co.uk; 4 Wireless Communications Research Group (WiCR), Loughborough University, Leicestershire LE11 3TU, UK; w.g.whittow@lboro.ac.uk (W.G.W.);

**Keywords:** antenna under test (AUT), electromagnetics (EM), far-field measurements, printed-log periodic dipole antenna (PLPDA), two-antenna gain measurement method, unmanned aerial vehicle (UAV)

## Abstract

To evaluate the performance of a printed log-periodic dipole antenna (PLPDA) in outdoor environments, we present unmanned aerial vehicle (UAV)-based antenna measurements conducted in the far-field region. Non-tethered UAV flight operations were achieved by configuring commercially available UAVs separately as a transmitter (TX) and as a receiver (RX). UAVs configured in non-tethered mode provide flexibility in terms of altitude maintained by the UAV from the ground level. The TX section of the UAV consists of a portable signal generator and a PLPDA configured to transmit signals with an output power of +15 dBm at 0.8 and 3.5 GHz. Similarly, the RX section of the UAV is equipped with a real-time spectrum analyzer and an identical PLPDA. Using these two UAVs in TX and RX modes, the radiation pattern of the PLPDA was obtained in the azimuth plane. Since two identical PLPDAs were used, the realized gain of the PLPDA is evaluated using the two-antenna gain method. The test scenario involved the TX UAV hovering at the center while the RX UAV followed a circular trajectory around it. A comparison between the UAV measurements, anechoic chamber measurements, and simulated data demonstrates good agreement, validating the reliability of the measurements.

## 1. Introduction

There has been a significant improvement in characterizing the performance of an antenna, starting with measurements in an elevated slant range environment to measurements in an anechoic chamber. However, measuring structurally large antennas operating at lower frequencies, such as 175 MHz, poses challenges due to the limited space available inside an anechoic chamber [1,2]. To address these challenges, portable antenna measurement systems (PAMS) have been developed to measure structurally large antennas [3].

Environmental conditions such as weather, humidity, radio frequency (RF) interference, and ground reflections can influence antenna measurements. Characterizing antenna performance in outdoor conditions can ensure that it meets the design specifications and that its interaction with the outdoor environment is quantified. To address these requirements, measurements can be performed using an antenna mounted on an airborne platform, such as a manned aircraft or an unmanned aerial vehicle (UAV) [4]. Several measurement strategies have been adopted for UAV-based antenna measurements in near- and far-field regions [5,6,7,8,9].

However, in these measurement techniques, the antenna under test (AUT) is placed at ground level, generally on a dielectric or wooden tripod at a maximum altitude of 3 m from the ground level [7,8,9,10], and the UAV follows a trajectory around the AUT. The measured radiation patterns of the AUT often suffer from ground reflections and scattering effects, making it challenging to analyze the antenna’s performance in terms of the realized gain, HPBW, and front-to-back ratio. To overcome these effects authors in [11] propose using a tethered UAV inside an anechoic chamber to characterize horn antennas. In a tethered mode, antenna mounted on the UAV are connected to the RF equipment at the ground level through cables. Although this approach overcomes the losses due to scattering and ground reflections, it introduces new restrictions in terms of the UAV flight trajectory. Furthermore, the propellers of UAVs can damage the absorbers within the chamber, creating a potential hazard.

**Figure 1:**
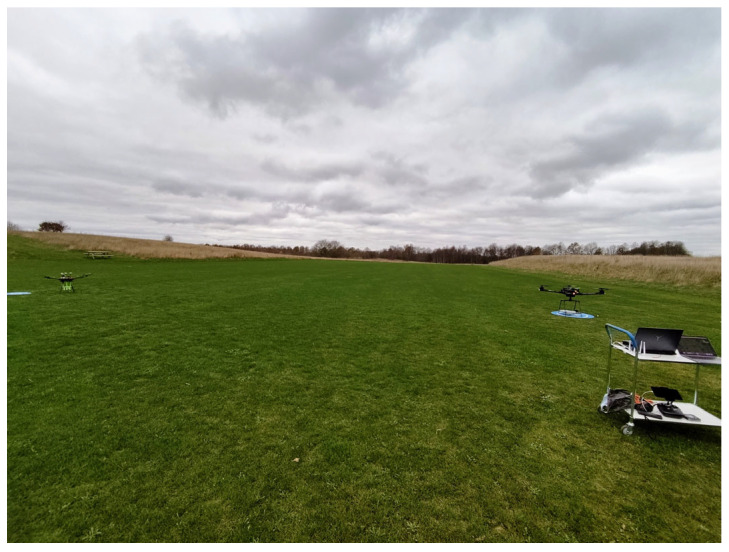
Two-antenna gain measurement setup.

To mitigate these limitations, we propose the use of two UAVs to measure the radiation pattern of the antenna in azimuth plane, as shown in Figure 1. In the proposed method, two UAVs are configured separately as a transmitter (TX) and receiver (RX) with an identical antenna, such as a printed log-periodic dipole antenna (PLPDA), mounted on TX and RX UAV. With the TX and RX UAVs configured in a non-tethered mode, both UAVs can maintain higher altitudes to overcome ground reflections. In a non-tethered mode, there are no physical connections between the ground-based equipment and equipment mounted on the UAV, allowing better maneuverability for the UAV. Non-tethered configuration allows the proposed UAV measurement solutions to be used for any kind of antenna, from simple dipoles and monopoles to the latest reconfigurable intelligent surfaces for beamforming applications [12,13,14]. Finally, the realized gain of the AUT is calculated using the two-antenna gain method during the UAV-based antenna measurements. Using the two-antenna gain method enables us to determine the realized gain of the antenna by accounting for all relevant loss mechanisms, including mismatch losses, substrate losses, and radiation losses. In this method, the propagation loss between the antennas is incorporated through the Friis transmission equation, ensuring that the calculated realized gain accurately reflects the true performance of the antenna under test. Similarly, the radiation patterns of the PLPDA in a stand-alone case, i.e., when the PLPDA enclosed in a fixture with foam is not mounted on the UAV, were also measured in an anechoic chamber with realized gain calculations using the two-antenna gain method. Measurements were conducted at 0.8 GHz and 3.5 GHz because they represent the key bands in modern wireless communications. Both frequencies are popularly used by mobile handsets, base station antennas, and IoT applications. While 0.8 GHz covers the LTE band, 3.5 GHz covers the primary 5G NR bands. Hence, the radiation patterns and gain of the antenna were measured at 0.8 GHz and 3.5 GHz to understand the antenna performance in outdoor environments. Furthermore, the technique can be used to measure 4G and 5G mobile communications base station antennas operating at the above-mentioned frequencies.

The novel contributions of this work are as follows:

- Evaluation of the performance of a PLPDA at various frequencies using two UAVs, thus avoiding ground reflections.
- Gain calculations of the PLPDA by applying the two-antenna gain method.
- Performance analysis of the PLPDA mounted on a UAV through S-parameter measurements.

In Section 2, we discuss the state-of-the-art in UAV-based antenna measurements. Section 3 provides the performance analysis of PLPDA. This involves improving the gains of the PLPDA at lower frequencies and overcoming the impedance mismatch experienced by the PLPDA enclosed in a fixture. We also discuss the performance of the PLPDA when mounted on the UAV, including an exploration of the electromagnetic (EM) coupling between the PLPDA and the UAV body components. The antenna measurement results obtained from UAV-based measurements are further elaborated in Section 4.

## 2. Related Work

Table 1 presents UAV-based antenna measurements for different scenarios. UAV-based measurements are beneficial for measuring complex and large structures deployed in the field, such as the Tianlai reflector antenna [15], Cassegrain dual reflector antenna, Square Kilometer Array (SKA) telescope antennas [16], Base Station Antennas (BASTA) [17], and broadcasting antennas [18]. Advances in UAV technology and improvements in battery life have enabled longer UAV flight times. UAVs are widely used to calibrate antennas deployed in the field [19,20]. Researchers have presented their work on measuring antennas in either the far-field (also known as Fraunhofer Zone) or near-field regions (also known as Fresnel Zone). An example of this is a UAV carrying a monopole to characterize an antenna operating in the 3 to 30 MHz range [21]. The AUT is a Nostradamus system consisting of 288 biconical antennas arranged along a 120° branch. The biconicals are omnidirectional, each covering a height of 7 m and a width of 6 m. To validate the accuracy and functionality of UAV-based near-field measurements for characterizing biconical antennas by the Office National d’Etudes et de Recherches Aérospatiales (ONERA) in [21], a UAV equipped with a monopole is used to measure another monopole that is 6 m high. The measured data was compared with simulated data. Based on these comparisons, it is evident that UAV-based near-field measurements offer accurate and cost-effective solutions for characterizing structurally large biconical antennas. Similarly, in [2], a UAV configured as a TX is used to measure antennas at 175 MHz. The measurement scenario consists of a dipole mounted on the UAV transmitting signals with an output power of +5 dBm. The AUT is the pre-aperture array verification system of the SKA. The AUT has an overall size of 9.2 m which is placed over a ground plane of 16 m diameter. UAV-based measurements are performed in the near-field region, and near-field to far-field transformations are performed. The far-field patterns obtained are compared with the simulated data. The comparison shows that the UAV-based measurements are in good agreement with the simulated data.

## 3. EM Performance of the PLPDA Mounted on UAV

### 3.1. Radiation Patterns of the PLPDA

**Figure 2:**
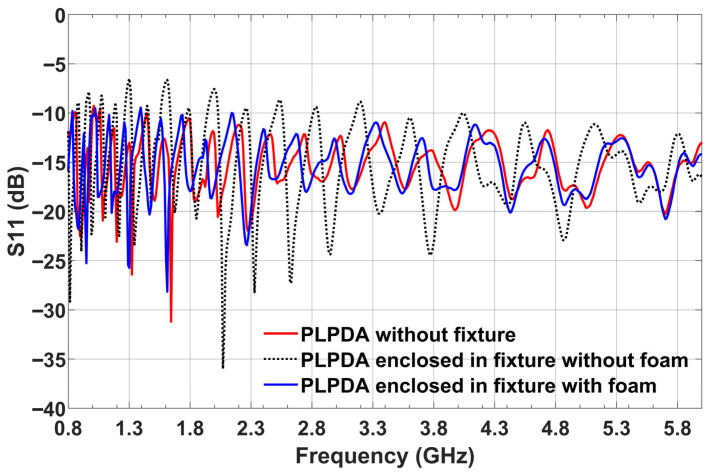
Measured S11 of PLPDA in an anechoic chamber.

**Figure 3:**
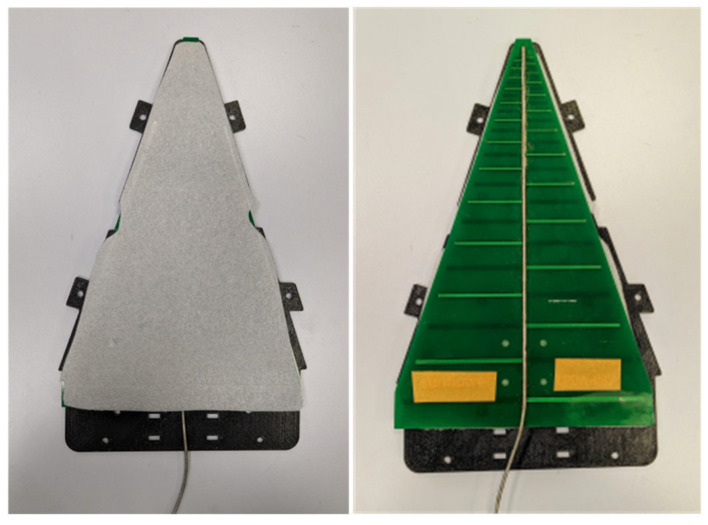
Foam material for dielectric loading.

**Figure 4:**
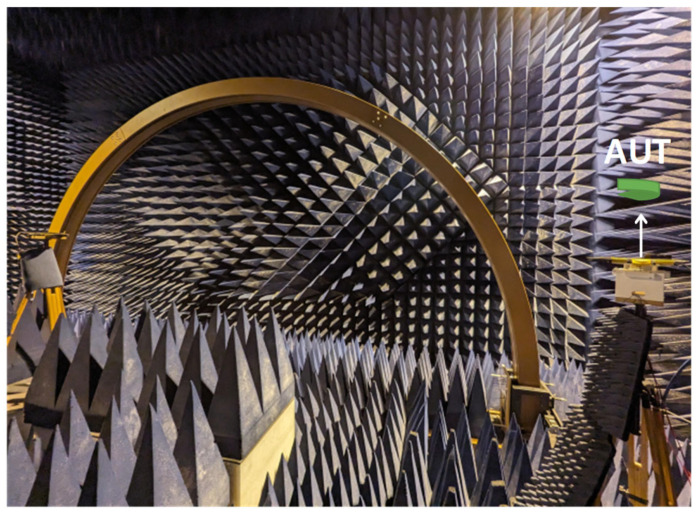
Measurements in an anechoic chamber (Az cut).

To perform UAV-based antenna measurements over a wide frequency range, wideband antennas with end-fire radiation patterns have several advantages. By using a wideband antenna, we can avoid changing the antenna on the UAV when there is a change in the frequency of operation of the AUT. Unlike antennas with an omnidirectional radiation pattern, antennas with end-fire radiation patterns have advantages in terms of avoiding EM coupling between the UAV body and the antenna mounted on the UAV. In our case, we used a PLPDA with a wide bandwidth [27,28] and a relatively flat realized gain of 6.5 dBi over a frequency range of 0.8 to 6 GHz. Due to their wide bandwidth and relatively flat realized gain, proposed UAV systems are suitable for conducting antenna measurements across various frequency bands. An average realized gain of 6.5 dBi across the frequency band enables reliable long-range communication, facilitating substantial separation distances between transmitting and receiving UAVs. The PLPDA used has dimensions of 250 mm × 170 mm, with a length of 250 mm, log-periodic antenna in printed version that could experience mechanical vibrations when the UAV is following a trajectory. To avoid these vibrations and for mounting the PLPDA on the UAV, we designed a fixture. The proposed fixture is fabricated using fused deposition modeling-based 3D printing and is made of poly-lactic acid (PLA) material. PLA is a low-loss material with a loss tangent of 0.01 at 2.45 GHz, electrical conductivity of 1.36 × 10^−3^ S/m and a dielectric constant of 2.5. To understand the effect of fixtures on the PLPDA performance, S11 parameters of the PLPDA enclosed in the fixture were measured. In Figure 2, we can observe the measured S11 parameters of the PLPDA without fixture and PLPDA enclosed in the fixture. From the measurements, it can be observed that there is an impedance mismatch across the operational frequency due to the fixture. To overcome the impedance mismatch, we propose dielectric loading. The proposed technique involves using foam material with a loss tangent of 0.0002 at 10 GHz, electrical conductivity of 1.11 × 10^−4^ S/m and a dielectric constant of 1.8 at 10 GHz. The foam material with a thickness of 5 mm is placed on one side of the PLPDA. In Figure 3, we can observe the foam material placed on the top of the PLPDA. The foam material, whose electric properties are close to the electric properties of air, creates an effective air layer between the FR4 substrate of PLPDA and the fixture. In Figure 2, the measured S11 parameters of PLPDA enclosed in a fixture with foam are overlapped with the other two cases. These comparisons show that the impedance mismatch observed due to the fixtures is now resolved. Furthermore, radiation patterns of the PLPDA without fixture and the PLPDA enclosed in a fixture with foam were measured in the azimuth plane. These measurements were performed in an anechoic chamber, as shown in Figure 4. The measurement procedure consists of two identical PLPDAs enclosed in a fixture with foam. The identical PLPDAs, one configured as a reference antenna and the other as AUT, are separated by 3 m (tip to tip). The reference antenna and AUT are connected to the VNA placed outside the chamber.

**Figure 5:**
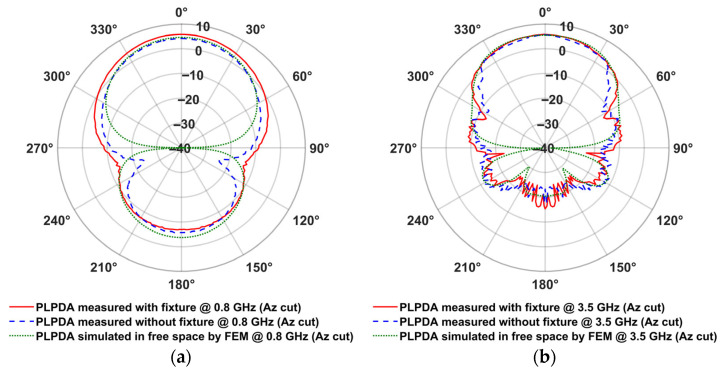
Measured gain of PLPDA with and without fixture in an anechoic chamber (Az cut) at (**a**) 0.8 GHz; (**b**) 3.5 GHz.

The measured radiation patterns for the PLPDA without fixture and PLPDA enclosed in a fixture with foam were compared with the simulated data of PLPDA without fixture, as described in Figure 5a,b [29]. The Finite Element Method (FEM) is used to carry out simulations. To perform antenna measurements in the far-field it is essential to calculate the required far-field distance. This distance is given by ${2D}^{2}/\lambda$, where *D* is the maximum dimension of the antenna and λ is its operating wavelength. The PLPDA measured consists of dipoles on either side of the FR4 substrate, with dimensions of 250 mm × 170 mm × 1 mm. With a maximum dimension of 250 mm and highest frequency of operation at 6 GHz, the far-field region would start at a distance greater than approximately 2.5 m, i.e., the separation distance between the two PLPDAs should be at least 2.5 m. By maintaining a separation distance of 3 m between the two identical PLPDAs in the anechoic chamber, the measurements are carried out in the far-field region. Conducting the measurements in the far-field allows for amplitude-only measurement, meaning that phase information is not always required.

**Figure 6:**
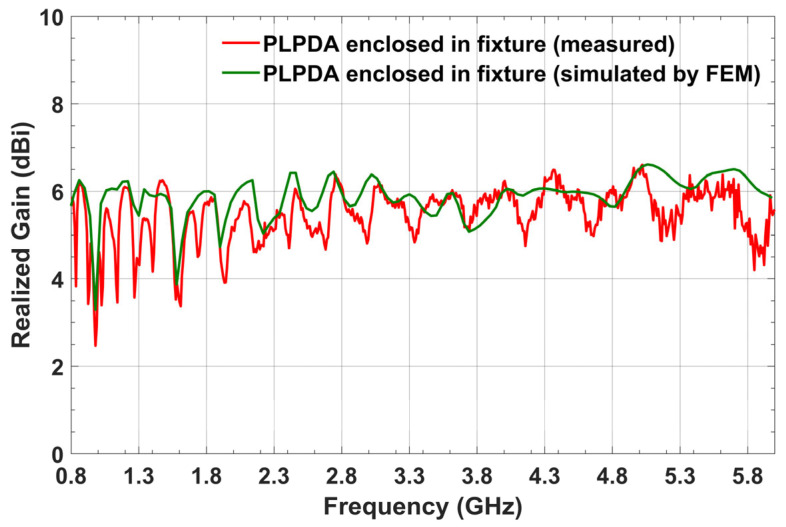
Measured and simulated realized gain of PLPDA enclosed in fixture with foam.

From the measurements shown in Figure 5a, for PLPDA enclosed in a fixture with foam, a realized gain of 5.8 dBi was obtained at 0.8 GHz. Similarly, at 3.5 GHz, a measured realized gain of 5.5 dBi was obtained. On the other hand, from measurements for a PLPDA without a fixture a realized gain of 4.2 dBi and 5.6 dBi were obtained at 0.8 and 3.5 GHz respectively. To evaluate the accuracy of the PLPDA simulations, the realized gain of PLPDA enclosed in a fixture with foam were measured from 0.8 to 6 GHz. These measured results are compared with the simulated results of PLPDA enclosed in fixture, as shown in Figure 6. Enclosing the PLPDA within the fixture and foam increases the effective dielectric constant around the antenna. This causes the resonance to shift to lower frequencies. As a result, the realized gain at 0.8 GHz is improved.

### 3.2. Performance Analysis of PLPDA on the UAV

Once the fixture for the PLPDA has been designed, the next critical phase involves mounting the PLPDA and RF equipment onto the UAV. It is worth noting that the carbon fiber material commonly used in UAV construction is highly conductive, potentially causing EM coupling with the PLPDA. To overcome the EM coupling between the UAV body and the PLPDA, it is important to mount the PLPDA at an appropriate location on the UAV. Before performing any measurements using the UAVs, it is imperative to assess the overall performance of the RF system. EM coupling can lead to electrical squints in the beam peak, reducing the system’s overall efficiency. Therefore, ensuring proper mounting and conducting a thorough assessment of the RF system’s performance beforehand are essential steps to optimize the accuracy and functionality of the PLPDA and RF equipment when mounted on the UAV. For this purpose, a portable vector network analyzer, the Lite VNA 64 [29], was used to measure the S11 parameters of the PLPDA mounted on the UAV. The initial placement of the PLPDA involves attaching the PLPDA to the base plate of the TX UAV. This base plate is located near the landing gear of the UAV.

**Figure 7:**
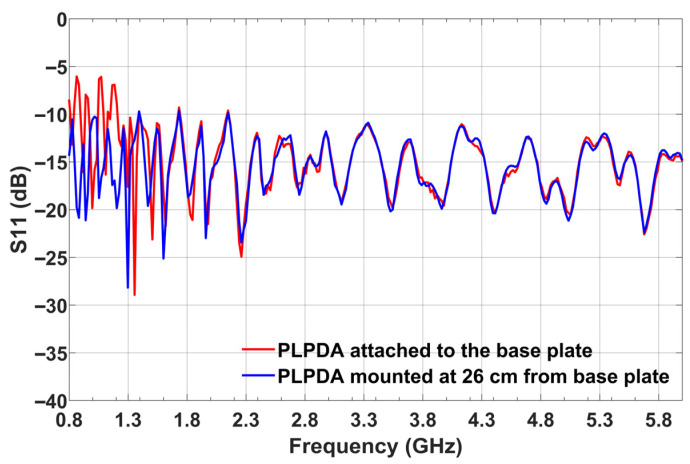
Measured S11 of PLPDA mounted on TX UAV.

Figure 7 illustrates the measured S11 data for the two cases: PLPDA attached to the base plate and PLPDA mounted 26 cm below the base plate. When the PLPDA is attached to the base plate, it can be observed that the PLPDA experiences an impedance mismatch up to 1.3 GHz. To overcome this mismatch, 3D-printed rectangular blocks made of PLA were used to move the PLPDA to an appropriate location (a location free from EM coupling). From the results described in Figure 7, it is evident that when the PLPDA is mounted 26 cm from the base plate of the UAV, the PLPDA is now well matched with a return loss better than 10 dB across the operating frequency band. In addition to considerations of electromagnetic performance, positioning the PLPDA in close proximity to the onboard UAV antennas may result in interference between the PLPDA (any antenna operating in GPS frequency bands) and the onboard UAV antennas. This interference can create substantial challenges for the remote controller at the ground level when operating the UAV and for establishing telemetry links. Considering these aspects, the PLPDA was mounted at the bottom of the UAV to overcome these issues. The impedance mismatch experienced by the PLPDA when attached to the UAV base plate could potentially lead to a reduction in the overall radiated power of the antenna. To understand the effect of impedance mismatch on the radiated power of the PLPDA, the TX UAV modeled in CST was simulated at 0.8 and 3.5 GHz. These simulations were performed when the PLPDA was attached to the base plate of the UAV (case 1) and when the PLPDA was mounted 26 cm from the base plate of the UAV (case 2) [30,31].

**Figure 8:**
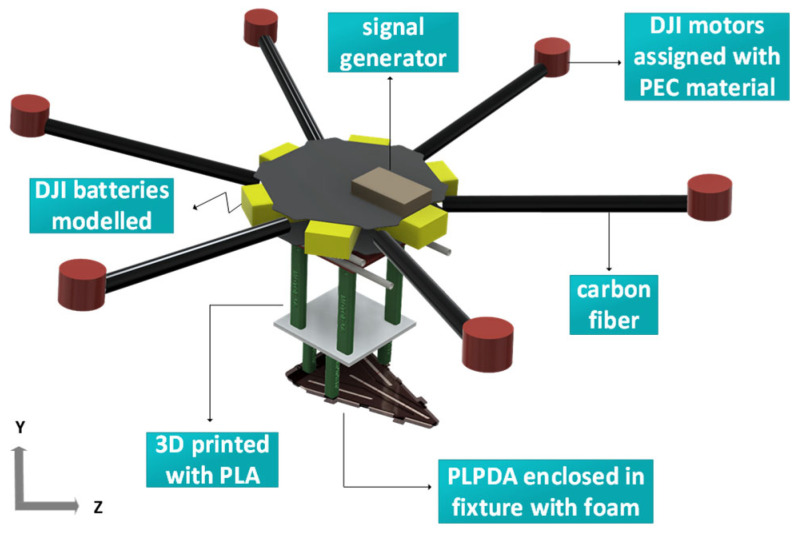
EM model of the TX UAV.

**Figure 9:**
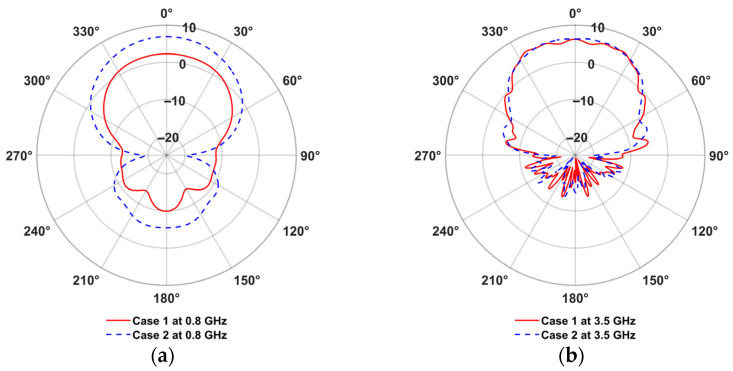
Simulated gains of PLPDA mounted on TX UAV in the azimuth plane at (**a**) 0.8 GHz; (**b**) 3.5 GHz.

To overcome the excessive computational efforts due to the electrically small plastic components, the TX UAV has been simplified, as shown in Figure 8. In the simplified EM version, propeller motors, batteries, base plate and signal generators were considered as perfect electric conductors (PEC). Other components, such as support plates, and arms, were modeled as carbon fiber. During simulations, an electrical conductivity of 1.213 × 10^−2^ s/m, a dielectric constant of 6.5 and a loss tangent of 0.089 at 2.45 GHz are considered for the carbon fiber [32]. For the FR4 material of the PLPDA, a dielectric constant of 4.3 and a loss tangent of 0.02 at 2.3 GHz were used. Simulations were performed using an i9-13900 KF processor @ 3 GHz (32-cores) and 128 GB of RAM, accelerated by an NVIDIA RTX4090 GPU with a CST acceleration token. With 241 million hexahedral mesh cells, the EM model of the UAV was simulated using the transient Finite Integration Technique. With these settings, it took around two hours to complete the simulations. As shown in Figure 9a, case 1 has a significant reduction in realized gain at 0.8 GHz compared with case 2, where the PLPDA is mounted 26 cm below the base plate. At 0.8 GHz, when the PLPDA is attached to the UAV base plate, a realized gain of 2.6 dBi is obtained, whereas a realized gain of 6.8 dBi is achieved when the PLPDA is mounted 26 cm below the base plate. On the other hand, from Figure 9b, it can be observed that at 3.5 GHz, for case 1 (PLPDA attached to the base plate) has several distortions in the radiation pattern due to the EM coupling between the UAV body and the PLPDA.

## 4. UAV-Based Antenna Measurements

**Figure 10:**
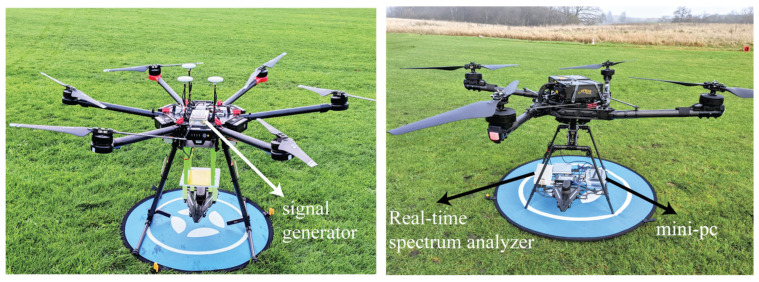
UAV configured as TX and RX (TX on left side and RX on right side).

After improving the performance of the PLPDA integrated with the UAV, outdoor antenna measurements were conducted using the UAVs. The UAV configured as a TX comprises a PLPDA enclosed in a fixture and a portable signal generator. The portable signal generator [33], including an expansion module, can generate signals with a maximum output power of +15 dBm. Measuring 113 mm × 70 mm × 25 mm in dimensions and weighing 205 g, it can generate Continuous Wave (CW) signals from 100 kHz to 6 GHz, with an output resolution of 0.25 dB. The PLPDA mounted on the UAV is connected to the proposed portable signal generator, as shown in Figure 10 (left side). The TX UAV is hovering at the center of the planned trajectory. The TX UAV is ensured to be stationary throughout the measurements with a total payload weight of 245 g. In contrast, Alta X UAV is configured as a RX to capture EM signals. All the necessary RF equipment, including the real-time spectrum analyzer [34,35] and identical PLPDA, are mounted on the RX UAV. The SA44B from Signal Hound is the real-time spectrum analyzer used in the measurements [36]. This real-time spectrum analyzer can detect signals with a maximum power level of up to +20 dBm across a frequency range of 150 kHz to 4.4 GHz [37]. Other components include a MeLE quieter 3c mini-PC and a power bank to power the mini-PC. In Figure 10 (right side), we can observe all components mounted on the RX UAV. With all this equipment, a total weight of 1.85 kg was observed at the RX UAV. The initial trajectory planning, and calculations were carried out using Alta X QGroundControl 4.4, and the results were then provided as input to the RX UAV through a ULog file.

The final circular trajectory consists of 24 waypoints. The waypoints are the GPS coordinate values at which the RX UAV travels and halt for 10 s at each point. During trajectory planning using QGroundControl 4.4 (software tool for trajectory planning), the GPS coordinates of each waypoint are assigned such that they correspond to the 15° orientation of the PLPDA with respect to the boresight of the PLPDA at the center of the trajectory. When the RX UAV follows the trajectory, received RF power is continuously logged. For synchronizing the timestamps between the UAV GPS and the real-time spectrum analyzer, the RX UAV halts at each waypoint. When the RX UAV launches from ground level, a remote connection is established to the mini-PC mounted on the UAV. All DSP operations performed by the real-time spectrum analyzer on the mini-PC can be controlled from a laptop at ground level. At this point, the Spike software [38] is used to log RF power values along with timestamps. The logged data consists of power values in dBm corresponding to the timestamps in an Excel file. The logged data and the timestamps logged by the GPS logger are used to analyze the radiation pattern in the azimuth plane.

**Figure 11:**
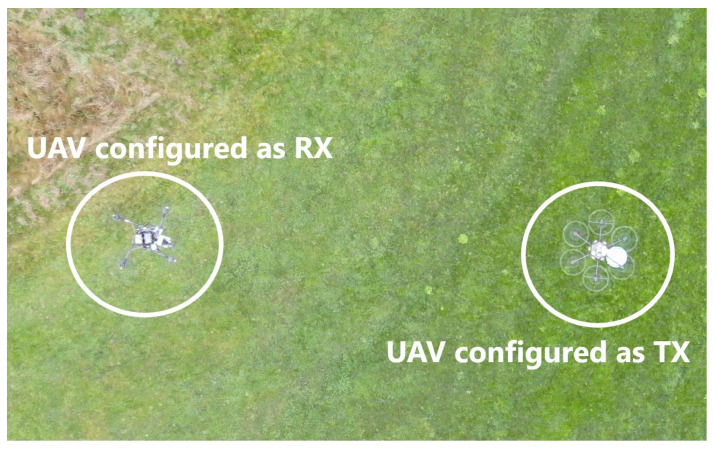
Aerial view of UAVs during the measurements.

In Figure 11 we can observe the aerial view of both UAVs, with the RX UAV following a circular trajectory and the UAV configured as a TX at the center of the trajectory. All the measurements were performed in Silkeborg EL & Svaev, a model club for electric vehicles and gliders in Silkeborg, Denmark [39]. During the measurements, it was observed that the environmental conditions were relatively harsh, with a temperature of −2 °C and a wind speed of 8 kmph. All the components mounted on the transmitter and receiver UAVs performed well under these weather conditions. This proves that all configured components are suitable for outdoor measurements under these weather conditions. During the trajectory, the GPS on the RX UAV was connected to more than 28 satellites, ensuring excellent positional accuracy for the UAV. For UAV measurements, aligning the UAVs is important to receive the maximum power from the UAV configured as RX. During the measurements, while one UAV remained stationary and hovered at a fixed position, the altitude of the second UAV was adjusted upward and downward in the elevation plane to determine the altitude at which the maximum received power occurred. Once the maximum received power was identified, the altitude of the second UAV was fixed at that level, and a circular trajectory was then executed [40,41].

**Figure 12:**
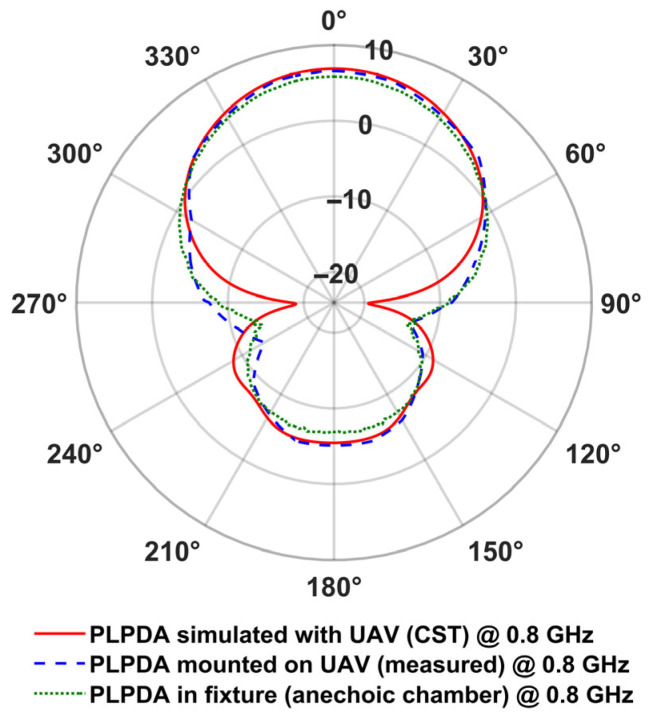
Measured and simulated realized gain of PLPDA at 0.8 GHz in the azimuth plane.

**Figure 13:**
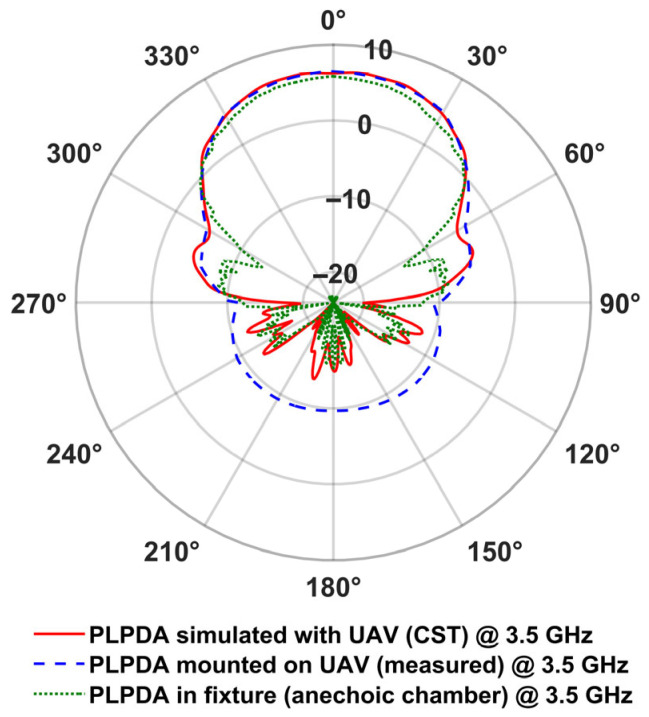
Measured and simulated realized gain of PLPDA at 3.5 GHz in the azimuth plane.

Once the RX UAV finishes its circular trajectory and returns to its designated home point, all the log files saved on the mini-PC are transferred to an external storage device. After obtaining the log files from the real-time spectrum analyzer and RX UAV GPS, the next task is synchronizing their timestamps. Synchronizing the timestamps between the real-time spectrum analyzer and the GPS on the UAV has been a major shortfall in UAV-based measurements. Many researchers have relied on a physical connection between the UAV and the ground equipment to achieve synchronization, which restricts them from reaching higher altitudes and limits their ability to conduct UAV measurements in diverse test scenarios [11]. A MATLAB script was developed to extract the timestamps from every waypoint. With the timestamps at every waypoint, power values corresponding to the start and stop times from the timestamps are averaged. These averaged power values correspond to the received power for every 15°. Using the received power values at every 15°, the Friis transmission equation is applied to calculate the gain of the AUT. The gain calculations are performed using the expression:

(1) $$G_{dBi}=\frac{1}{2}\left[20\;{log}_{10}\left(\frac{4\pi R}{\lambda }\right)\;+10\;{log}_{10}\left(\frac{P_{r}}{P_{t}}\right)\right],$$

where *G_dBi_* is the realized gain of the antenna in *dBi*, *R* denotes the distance between the TX and RX, *λ* is the wavelength, *P_r_* is the received power in milliwatts, and *P_t_* is the transmitted power also in milliwatts. Figure 12 and Figure 13 depict the realized gain of the PLPDA measured in the presence of the UAV at 0.8 GHz and 3.5 GHz respectively. The measured radiation patterns of the AUT are compared with the simulated data.

As described in Table 2, from the UAV-based measurements, it is observed that a realized gain of 6.5 dBi was obtained at both 0.8 and 3.5 GHz. On the other hand, from the simulations (PLPDA mounted on the UAV), realized gains of 6.8 dBi and 6.2 dBi were obtained at 0.8 and 3.5 GHz, respectively. From these results, and from the radiation patterns measured using the proposed two UAV method, it is evident that ground reflections can be mitigated, i.e., reflections that are typically observed when antennas are measured outdoors at lower altitude [37].

In terms of cost, the proposed solution is highly cost-effective and offers substantial savings. The overall cost depends on the UAV platform selected for the measurements. As presented in Table 3, several commercially available UAV options are suitable, and users may select a platform based on the required payload, i.e., the weight of the measurement equipment. In our experiments, as discussed earlier, we used the DJI Matrice 600 Pro and the Freefly Alta X, that cost around USD 10–20 k each. In Table 3, the maximum payload capacity for each UAV model and the corresponding range of UAV flight times are presented, since flight time depends on several factors such as the total payload on the UAV, the type of trajectory planned, and external environmental conditions including temperature and wind pressure. For the RF equipment the total cost is around USD 2–3 k. Thus, the total cost of the proposed setup is around USD 30 k to 40 k. On the other hand, a traditional antenna measurements anechoic chamber would cost anywhere from USD 500 k to several million depending on the size and equipment of the anechoic chamber. Therefore, the UAV-based approach is significantly more cost-effective, although not as accurate as a state-of-the art anechoic chamber including a near-to-far field transformation setup or a CATR (Compact Antenna Test Range).

The accuracy of UAV-based antenna measurements depends on several factors, including the precision of RF instruments, UAV vibrations, GPS positioning accuracy, and environmental conditions. Modern UAVs typically employ high-precision positioning systems such as real-time kinematics (RTK), differential RTK (D-RTK), or differential global navigation satellite systems (GNSS), with the specific technique selected based on the measurement frequency and required positioning accuracy. Environmental effects, particularly wind, can introduce small deviations in the UAV trajectory, typically resulting in less than 0.4° angular deviation. Minor trajectory shifts may lead to variations of approximately 0.02–0.1 dB in the measured radiation pattern, while changes in UAV orientation can introduce an uncertainty of about ±2%, corresponding to a very small measurement variation [1].

## 5. Conclusions

The utilization of a wideband antenna such as a PLPDA is suitable for UAV-based antenna measurements. The utilized PLPDA experiences impedance mismatch when it is directly enclosed in fixtures. This issue has been addressed by adopting the dielectric loading technique, which involves using foam material inside the PLPDA fixture. Critical challenges, such as mitigating EM coupling between the PLPDA and the UAV body, were addressed through measurements and simulations. Cost-effective and green solutions like 3D-printed components were used to overcome EM coupling. To overcome the ground reflections, a novel technique of using two UAVs is described. This involves configuring the UAVs in TX and RX mode. These UAVs are configured in a non-tethered mode, thus providing better flexibility for the UAV flight. The successful measurement of the PLPDA radiation patterns at 0.8 GHz and 3.5 GHz in the azimuth plane resulted in HPBWs of 75° and 70°, respectively. The two-antenna gain measurement methods were used to perform gain measurements, resulting in a realized gain of 6.5 dBi at both 0.8 and 3.5 GHz. These results unveil new possibilities for performing antenna measurements with increased flexibility and precision, emphasizing the potential of UAV-based methods for future research and application in the field of antenna technology.

## Figures and Tables

**Table 1:** Overview of State-of-the-Art UAV-based antenna measurement techniques.

Reference	Frequency of Operation	Measurement Region	Antenna Under Test	Antenna Mounted on UAV
[8]	3 GHz	Far-field	Phased Array antenna	Microstrip patch antenna
[9]	14.5 GHz	Far-field	Reflector antenna	Dual-polarized quad ridge horn antenna
[10]	2.45 GHz	Near-field	Double-ridge horn	Vivaldi antenna
[11]	1.8 and 2.7 GHz	Near-field	Pyramidal Horn	Dual-polarized microstrip patch antenna
[15]	730 MHz	Far-field	Tianlai reflector antenna	Dipole antenna
[22]	470–860 MHz	Far-field	Broadcasting antennas	Log-periodic antenna
[23]	50 MHz	Near-field	Aperture Array Verification System for Square Kilometer Array Telescope	Dipole antenna
[24]	2.1 GHz	Near-field	Cassegrain Dual Reflector antenna	Dual-polarized Vivaldi
[25]	4.65 GHz	Near-field	Circularly polarized horn antenna	Printed version of the monopole antenna
[26]	20 MHz	Near-field	Ground Plane antenna	Dipole antenna

**Table 2:** Comparison between simulations and measurements at 0.8 and 3.5 GHz.

Parameter	PLPDA Mounted on UAV and Measured with two UAV Method/Simulated with CST	PLPDA Alone Measured in Anechoic Chamber/Simulated with CST
Realized Gain (@ 0.8 GHz)	6.5/6.8 dBi	5.8/5.8 dBi
HPBW (@ 0.8 GHz)	75/73°	75/71°
Realized Gain (@ 3.5 GHz)	6.5/6.2 dBi	5.8/5.5 dBi
HPBW (@ 3.5 GHz)	70/71°	76/78°

**Table 3:** Commercially available UAVs.

UAV Model	Payload Capabilities	Flight Time Considering Payload
DJI Phantom 4	1 kg	18 to 25 min
DJI Matrice 300 RTK	2.7 kg	30 to 40 min
DJI Matrice 400 (latest model)	6 kg	30 to 44 min
DJI Matrice 600 Pro	6 kg	18 to 32 min
Freefly Alta X	15 kg	15 to 35 min
DJI Flycart 30	30 kg	18 to 29 min

## Data Availability

Data are contained within the article.
